# Therapeutic effect of *Aegle marmelos* fruit extract against DMBA induced breast cancer in rats

**DOI:** 10.1038/s41598-020-72935-2

**Published:** 2020-10-22

**Authors:** Vivek Akhouri, Manorma Kumari, Arun Kumar

**Affiliations:** 1 Anugrah Narayan College, Patna, Bihar India; 2 Research Centre, Mahavir Cancer Sansthan and Research Centre, Patna, Bihar 801505 India

**Keywords:** Biochemistry, Cancer, Cell biology, Drug discovery, Zoology, Oncology

## Abstract

Breast cancer is among most common form of cancer worldwide. It is also the major cause of death in female cancer patient around the world. Despite various therapeutic measures, it remains associated with high mortality rate. *Aegle marmelos* (L.) Correa has been extensively used in Indian medicine system *Ayurveda*, due to its various medicinal properties. However, there are very limited reports regarding its anticancer activity. Thus, the present research work has been aimed to study the anticancer activity of *Aegle marmelos* fruit extract on 7,12-dimethylbenz(a)anthracene (DMBA) induced breast cancer in rats. Female Charles Foster rats, 55–60 days old weighing around (150 ± 10 g) were used for the study and were induced DMBA (20 mg/mL dissolved in Olive oil) orally. After the development of breast tumors (about 0.5 cm), the rats were treated with *Aegle marmelos* ethanolic fruit pulp extract (200 mg/kg b.w./day) orally for 5 weeks and then volume of tumor was measured. *Aegle marmelos* treatment showed significantly reduced mammary tumor volume (*P* < 0.05), along with significant reduction (*P* < 0.0001) in the different serum biomarkers such as TNF-α level, serum malondialdehyde (MDA) level and glucose levels. Significant (*P* < 0.0001) improvement in both, the kidney and liver serum biomarker parameters were also observed after the treatment with *Aegle marmelos* ethanolic fruit pulp extract. From the entire study, taking everything into account it can be interpreted that *Aegle marmelos* ethanolic fruit pulp extract possesses anti-proliferative activity by suppressing the progression of breast tumors in rat model. The plant extract also possesses hepato-renal protective effect. Hence, it can be targeted as novel and safe anti-cancer drug against breast cancer.

## Introduction

Cancer is a multifactorial genetic disease, causing uncontrolled division and proliferation of abnormal cells in the body and the spread of these abnormal cells into the other body tissues. Cancer is the second most leading cause of mortality around the world. Breast cancer is the very often detected cancer among women worldwide and is also the dominant cause of death due to cancer in over 100 countries^1^. According to GLOBOCAN 2018, breast cancer is now known to be frequently diagnosed cancer and is most usual cause of cancer related death among women in India, with an estimated 27.7% (1,62,468) of all new cancer cases among women, and 23.45% (87,090) of cancer related death among women in 2018^2^. The etiology of breast cancer includes age, hereditary factor, reproductive factors, prolonging exposure to estrogen, lack of breastfeeding and other lifestyle associated factors. Besides these risk factors there are some environmental factors as well, which contributes in the development of breast cancer.

The factors like cigarette smoke, grilled or barbecued and smoked meat, non-metallic air toxics, along with numerous other lethal air pollutants especially polycyclic aromatic hydrocarbons (PAHs) increases the risk of breast cancer^3–6^. In the recent times, for the better yield of crops, pesticides are widely used by the farmers in the agricultural practices. These pesticides through the food chain has reached human body as xenoestrogens causing hormonal imbalance especially the estrogen levels in women. These elevations for longer period are responsible for causing disease like breast cancer^7–10^.

The polycyclic aromatic hydrocarbon (PAHs) are produced due to incomplete combustion of fossil fuel and other carbonaceous materials. These PAHs are released from the exhaust of industries, automobiles, fuels of biomass like burning coal for cooking in rural areas, tobacco smoke and forest fire^11^. Among many PAHs, 7,12‑dimethylbenz(a)anthracene (DMBA) is a chemical carcinogen widely used to induce mammary carcinogenesis in rats^12,13^. Although, liver is the primary organ, where chemical carcinogens are metabolically deposited and activated. Some extrahepatic tissues like mammary glands are also responsible for the deposition and activation of hydrophobic compounds like DMBA. Through a series of reactions by cytochrome p450 enzymes, DMBA is converted into DMBA-3,4-diol-1,2-epoxide (DMBA-DE) and the ultimate carcinogen interacts with DNA to form adducts which is responsible for carcinogenicity and mutagenicity^14^. DMBA carcinogenesis process involves disruption and interruption of tissue redox balance, creating oxidative stress which is liable for biochemical and pathophysiological disturbances in rats^15,16^. The reactive oxygen species formed cause cellular damage by lipid peroxidation resulting in cellular and subcellular changes.

Although, a number of anticancer drugs have been discovered, but apart from being expensive they have some serious side effects as well. So, it is important to develop safe, effective and economical treatment of the disease. Plethora of medicinal plants have attracted attention among the scientific communities, for its therapeutic efficacies against a number of diseases including cancer. *Aegle marmelos* commonly known as “bael or wood apple” is a member of family Rutaceae. The tree is native to dry forest on hill and plain of Indian subcontinent, Cambodia, Laos, Thailand and Vietnam. In Indian medicine system *Ayurveda*, it has very important role in controlling the diseases caused in the gastrointestinal tract^17^. Furthermore, it has immunomodulatory as well as antibiotic effect. Various parts of the *Aegle marmelos* such as root, bark, leaves and fruits are used in Indian system of medicine for its numerous medicinal properties like antioxidant, antibacterial, antifungal, antidiarrheal, antidiabetic, antiproliferative, cytoprotective, hepatoprotective, fertility booster, analgesic, antiarthritis, contractile, antihyperlipidemic, cardioprotective, radioprotective, anticancer, antiviral, antiulcer, immunomodulatory and wound healing properties^17,18^. The phytochemical studies have shown that the *Aegle marmelos* fruit contains several classes of phytochemical compounds like carotenoids, phenolics, flavonoids, alkaloids, tannins, terpenoids, coumarins, steroids, saponins, lignins, phlobatannins, inulin and cardiac glycosides^19,20^. The flavonoids, tannins and phenolic compounds, that act as a primary antioxidant and free radical scavengers are found to be maximum in the alcoholic extract of the fruit pulp^20,21^. The phytochemicals such as aegeline, aegelenine, marmelin, o-methyl halfordinol, alloimperatorin, furocoumarins, psoralen, o-isopentenyl halfordinol, marmelosin, umbelliferone and scopoletin are the coumarins present in the fruit pulp of the *Aegle marmelos*^22,23^. *Aegle marmelos* leaf extract is found to be very effective against various tumor cell lines including breast cancer cell line MCF7 and MDA-MB-231 and it also has antineoplastic effects on the Ehrlich ascites carcinoma in Swiss albino mice^24,25^. The ethanolic fruit extract also shows cytotoxic activity against SKBR3 human breast adenocarcinoma cell line^26^. The antitumor potentiality of *Aegle marmelos* bark extract on DMBA induced papilloma in Swiss albino mice has also been reported^27^. Therefore, the present study aims to investigate the therapeutic potential of *Aegle marmelos* ethanolic fruit pulp extract against DMBA induced mammary cancer in Charles Foster rats.

## Materials and methods

### Chemicals and reagents

DMBA (7,12‑dimethylbenz[a]antheracene) manufactured by Sigma-Aldrich, USA, Product number D3254-1G, (CAS Number: 57-97-6), Lot# SLBX1136, P code: 1002660800 was purchased from the Scientific chemical store of Patna, Bihar, India. All the other solvents and chemicals used were of analytical grade 99%.

### Preparation of *Aegle marmelos* fruit pulp ethanolic extract

The *Aegle marmelos* fruit was collected from the tree present in the Anugrah Narayan College (A. N. College), Patna, Bihar and was identified by Prof. Manorma Kumari, Department of Botany, A. N. College Patna, Bihar, India. The fibres and seeds from *Aegle marmelos* fruit were removed and the pulp was extracted and was dried in incubator at 37 °C temperature. The dried pulp was grinded to fine powder which was further soaked in absolute ethanol for 48 h, and finally extracted with absolute ethanol using Rota Vapour apparatus. The LD_50_ dose of the ethanolic extract was found to be 2500 mg/kg body weight. The final dose as 1/10th dose of LD_50_ was calculated and titrated to 200 mg/kg body weight.

### Animals

Female Charles Foster strain rats (12 female) were provided by the animal house of Mahavir Cancer Sansthan and Research Centre, Patna, India (CPCSEA Registration no. 1129/bc/07/CPCSEA). All animal experiments were carried out (as per the guidelines and regulations) in accordance with Committee for the Purpose of Control and Supervision of Experiments on Animals (CPCSEA), New Delhi. The experimental work was approved by the Institutional Animal Ethics Committee (IAEC) with IAEC No. 2017/1G-10/08/17. Diet including food and water to rats were given *ad libitum* (prepared by laboratory itself)*.* Rats were acclimatized for 7 days before the start of the experimental work. These experimental rats were housed in standard polypropylene cages having 2 animals in each cage and they were randomly distributed into control and treated groups. The mean room temperature of the animal house was maintained at (22 ± 2 °C) for the rats with 12 h light/dark cycle.

### Experimental design

Animals (12 female, Charles Foster strain Rats), aged 55–60 days, weighing (150 ± 10 g) were classified into 3 groups of 4 animals each.

- Group I—Control.
- Group II—DMBA treated-DMBA induced rats only.
- Group III—*Aegle marmelos* treated-DMBA induced rats treated with *Aegle marmelos* ethanolic pulp extract (200 mg/kg body weight per day) for 5 weeks after tumor development (about 0.5 cm).

After the completion of dosing, rats were anaesthetized by diethyl ether and sacrificed in the diestrous phase of the estrous cycle. Blood samples were collected through the orbital puncture of the experimental rats. Serum were separated for biochemical tests, lipid peroxidation estimation, TNF alpha test, estrogen and progesterone hormonal analysis. Tissues of breast were fixed in 10 percent formalin for the histopathological studies.

### Tumor induction

Mammary gland tumors were induced in 55 days old female Charles foster rats weighing (150 ± 10 g). Freshly prepared single dose of 20 mg/mL of DMBA (7,12‑dimethylbenz(a)anthracene) diluted in olive oil was given intragastrically by Gavage method to the rats^28^. All the 8 female rats were aged 55 days weighing (150 ± 10 g). Rats were palpated weekly starting from 4th week after DMBA administration, to check for the tumor appearance. The first tumor appeared in the 16th week, after administration of DMBA while by 18th week tumor appeared in all the 8 rats.

### Measurements of mammary tumor volume

Mammary tumors were measured through Vernier calliper scale. Tumor volume (V) was calculated as V(cm^3^) = (L × B^2^)/2, where L (large diameter), and B (small diameter) are perpendicular, stated in centimetres (cm).

### Biochemical assay

Biochemical analysis were performed by the standard kit process (Coral crest) through (UV–Vis) spectrophotometer (UV-10, Thermo Scientific, USA). The serum glucose levels were estimated through GOD/POD method by Tinder^29^. The Liver Function Test as Serum Glutamic Pyruvate Transaminase (SGPT) and Serum Glutamic Oxaloacetate Transaminase (SGOT) were measured according to the method by Reitman and Frankel^30^, Alkaline Phosphate (ALP) by method Kind and King^31^, total bilirubin by method Jendrassik and Grofs^32^, while albumin levels measured by Dumas et al.^33^. The Kidney Function Test (KFT) were analysed through urea by Berthelot; Fawcett and Scott^34,35^, creatinine by Bones and Tausky^36^, and uric acid by Fossati and Prencipe^37^.

### Lipid peroxidation (LPO)

Thiobarbituric acid reactive substances (TBARS), as a marker of LPO, were evaluated through the double heating method^38^ based on the principle of spectrophotometric measurement of colour reproduced during the reaction to thiobarbituric acid (TBA) with malondialdehyde (MDA). For this study, 2.5 mL of 10% solution of Trichloroacetic acid (TCA) were mixed with 0.5 mL serum in a centrifuge tube and heated in the water bath at 90 °C for 15 min. After cooling at room temperature, the mixture were further allowed to centrifuge at 3000 rpm for 10 min, and 2 mL supernatant was mixed with 1 mL of 0.675% TBA solution in a test tube which was further heated in water bath at 90 °C for 15 min and left for cooling at the room temperature. Thereafter, further absorbance was measured by UV–Vis spectrophotometer (Thermo Scientific UV-10 USA) at 532 nm**.**

### Hormonal assay

Hormonal assessment was done using the ELISA method. The estradiol and progesterone ELISA kit was manufactured by Monobind Inc. 100 North Pointe Drive, Lake Forest, CA 92630 USA. Estradiol (Lot No. EIA-49K2I8) and progesterone (Lot No. EIA-48K2E8) levels were measured according to the manufacturer’s instructions and Saunders, 1994^39^.

### Estradiol

The normal estradiol range was calibrated and 25 µL of serum samples were taken in the microwell plate. Firstly, 50 µL estradiol biotin reagent was taken and then added into each microwell. The microwell plate was then gently mixed for 20–30 s and incubated at room temperature for 30 min. The 50 µL of estradiol enzyme reagent was added to each well and was again mixed gently for another 20–30 s. The plate was then covered and incubated at room temperature for 90 min. The content of the microwell plate was then discarded and was washed 3 times by 350 µL of wash buffer and blotted. Then 100 µL of substrate solution was then added to each well and incubated at room temperature for another 20 min. Finally, 50 µL of stop solution was then added to each well and was mixed gently. Absorbance was read at 450 nm (using a reference wavelength of 620–630 nm) through Merck ELISA reader in pg/mL value.

### Progesterone

The normal progesterone range was calibrated and 25 µL of serum samples were taken in the microwell plate. Firstly, 50 µL progesterone enzyme reagent was taken and then added into each microwell. The microwell plate was gently mixed for 20–30 s and then 50 µL progesterone biotin reagent was added into each well. The microwell plate was again mixed gently for 20–30 s and then covered and incubated for 60 min at room temperature. The content of the microwell plate was then discarded and was washed 3 times by 350 µL of wash buffer and blotted. The 100 µL of substrate solution was added to each well and incubated at room temperature for 20 min. Finally, 50 µL of stop solution was then added to each well and was mixed gently. Absorbance was read at 450 nm (using a reference wavelength of 620–630 nm) through Merck ELISA reader in ng/mL value.

### Tumour necrosis factor-alpha (TNF-α) assay

The serum TNF-α assessment was done using ELISA method. For the present study, rat TNF-α ELISA kit [manufactured by Diaclone, France (Cat. No. 872.010.001)] was used. The serum TNF-α level was measured according to the manufacturer’s instructions. First of all, the microwell plate was coated with the capture antibody. After that 100 µL of standard diluents and 100 µL serum were added to the appropriate wells. Then 50 µL of diluted detection antibody was added to each well and incubated at room temperature for 3 h and covered properly. The content was then discarded and washed 3 times with 300 µL of washing solution. The 100 µL of streptavidin-HPR solution was then added to the each well and again incubated at room temperature for 30 min and covered properly. The content was then again discarded and washed 3 times with 300 µL of washing solution. The 100 µL of TMB substrate solution was then added to each well and incubated in dark for 5–15 min at room temperature while covered. The 100 µL of stop reagent was then added to each well. Absorbance was read at 450 nm (using a reference wavelength of 620–630 nm) through Merck ELISA reader in pg/mL value.

### Statistical analysis

Results are presented as Mean ± Standard Error Mean (SEM). The significance between DMBA treated and *Aegle marmelos* treated group for tumor volume were analysed by Two-way Analysis of Variance (ANOVA) using time & drug as the two factors. The significance between control and treated group for all other parameters were analysed by One-way Analysis of Variance (ANOVA) followed by Tukey's multiple comparison test. The value *P* < 0.05 was considered statistically significant. The analysis were done using GraphPad Prism 5 Program (GraphPad Software, Inc., San Diego, USA).

### Histopathological study

Small pieces of breast tissues were fixed into 10% formalin for 24 h. The tissues were then dehydrated through ethanol and were embedded into paraffin. Sections of 5 µm were cut and stained with haematoxylin and eosin for histopathological investigation under light microscope.

### Ethical approval

All applicable international, national, and/or institutional guidelines for the care and use of animals were followed. The experimental work was approved by the Institutional Animal Ethics Committee (IAEC) with IAEC No. 2017/1G-10/08/17 of Mahavir Cancer Sansthan and Research Centre, Patna, India (CPCSEA Registration no. 1129/bc/07/CPCSEA). All animal experiments were carried out (as per the guidelines and regulations) in accordance with Committee for the Purpose of Control and Supervision of Experiments on Animals (CPCSEA), New Delhi. This article does not contain any studies with human participants performed by any of the authors.

### Informed consent

Not applicable.

## Results

### Morbidity and mortality

**Figure 1:**
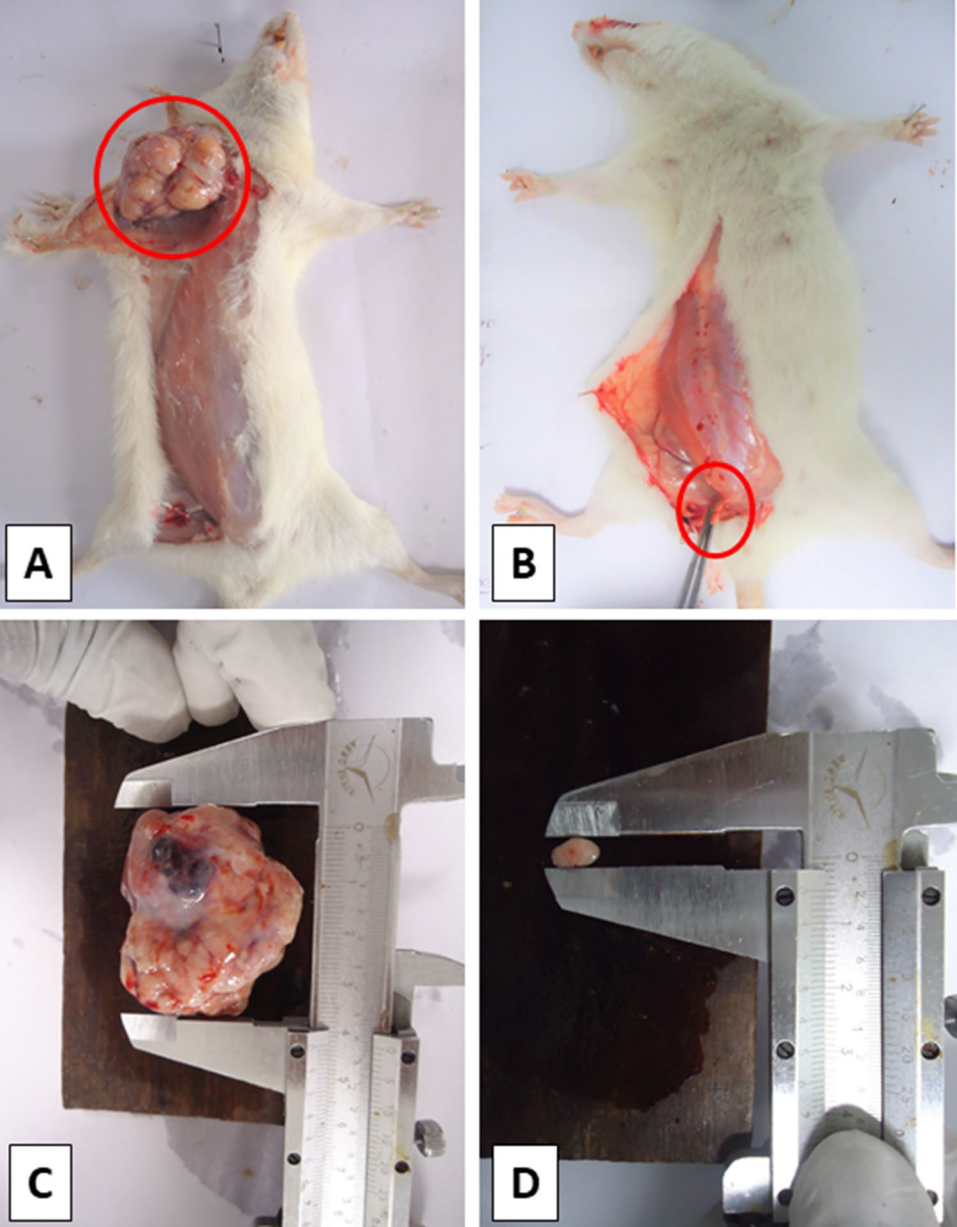
Gross photographs of rat mammary tumor of DMBA treated group (**A**,**C**) and *Aegle marmelos* treated group (**B**,**D**).

In DMBA treated group, tumor appeared in each of the four rats in mammary teat numbers 1, 3, 4 and 5. In the rest four rats in teat numbers 3, 4, 5 and 6, there was significantly very slow tumor progression in *Aegle marmelos* treated group. No mortality was observed in any of the studied groups. Figure 1 shows gross photographs of DMBA treated group and *Aegle marmelos* treated group.

### Effect on tumor volume

**Figure 2:**
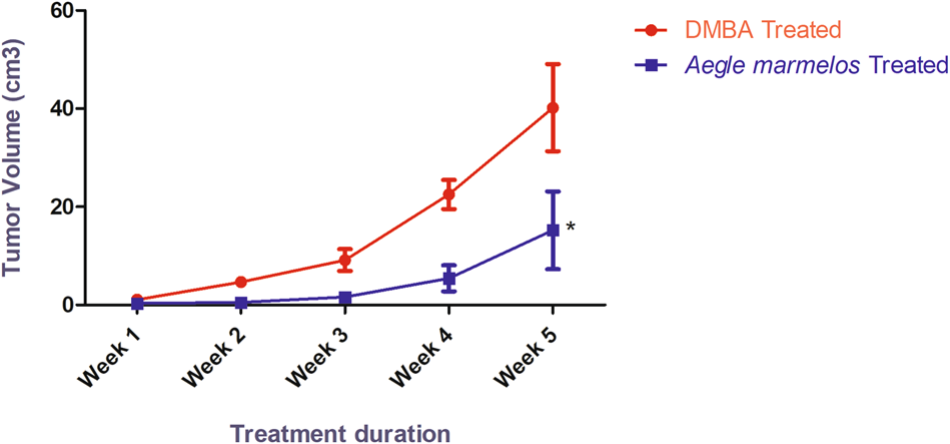
Effect of different treatments on tumor volume in the studied groups (n = 4, Significant *P* < 0.05, values are expressed as mean ± SEM).

With increasing time duration, tumor volume increased in both, the DMBA treated group and *Aegle marmelos* treated group. However, after the completion of the treatment, a significant decrease (*P* < 0.05) in the mammary tumor volume of the group that received *Aegle marmelos* ethanolic fruit pulp extract treatment after DMBA administration was observed in comparison to the group that received DMBA alone (Fig. 2).

### Effect on lipid peroxidation (LPO)

**Figure 3:**
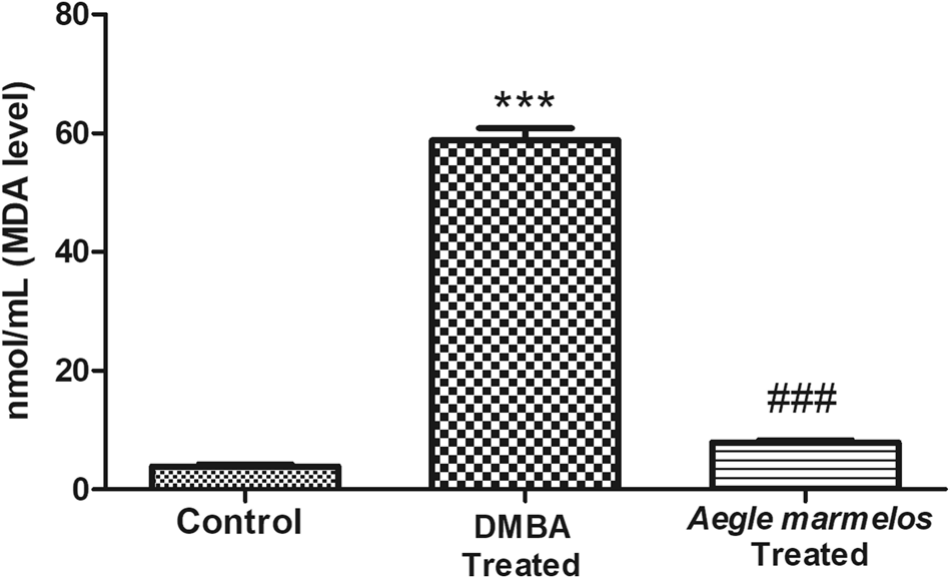
Effect of different treatments on lipid peroxidation level in the studied groups (n = 4, Significant ***P < 0.0001 compared with control group, ^###^*P* < 0.0001 compared with DMBA treated group, values are expressed as mean ± SEM).

The level of malondialdehyde (MDA) which is a marker of lipid peroxidation was also found significantly (*P* < 0.0001) higher in DMBA treated group as compared to the control group. However, after *Aegle marmelos* ethanolic fruit pulp extract administration, the MDA level reduced significantly (*P* < 0.0001) as compared to the DMBA treated group (Fig. 3).

### Effect on estradiol and progesterone hormone

**Figure 4:**
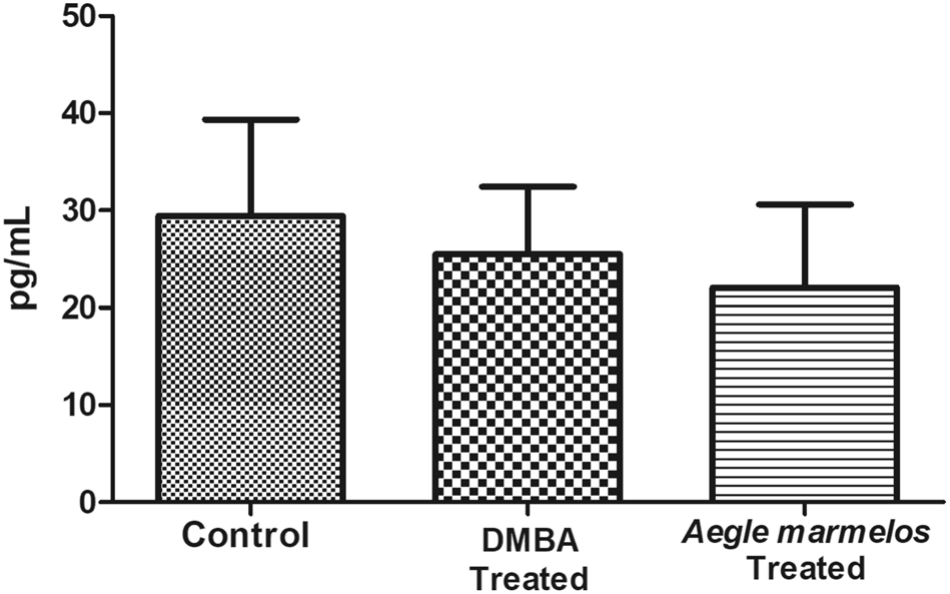
Effect of different treatments on estradiol level in the studied groups (n = 4, *P* > 0.05 (NS), values are expressed as mean ± SEM).

**Figure 5:**
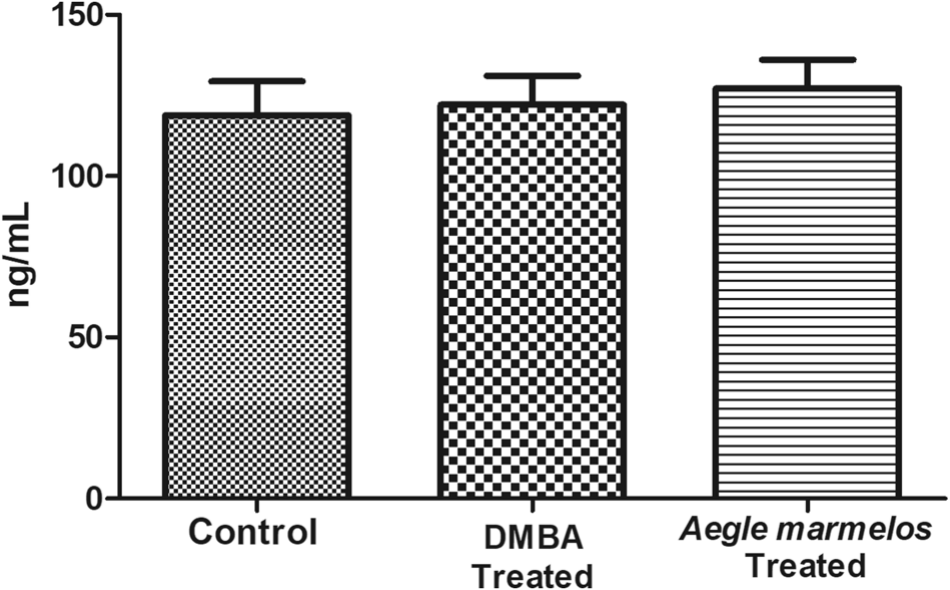
Effect of different treatments on progesterone level in the studied groups (n = 4, *P* > 0.05 (NS) values are expressed as mean ± SEM).

There was no significant changes observed in the estradiol and progesterone hormone levels between control and DMBA treated group in the diestrous phase of the estrous cycle (Figs. 4, 5).

### Effect on glucose level

**Figure 6:**
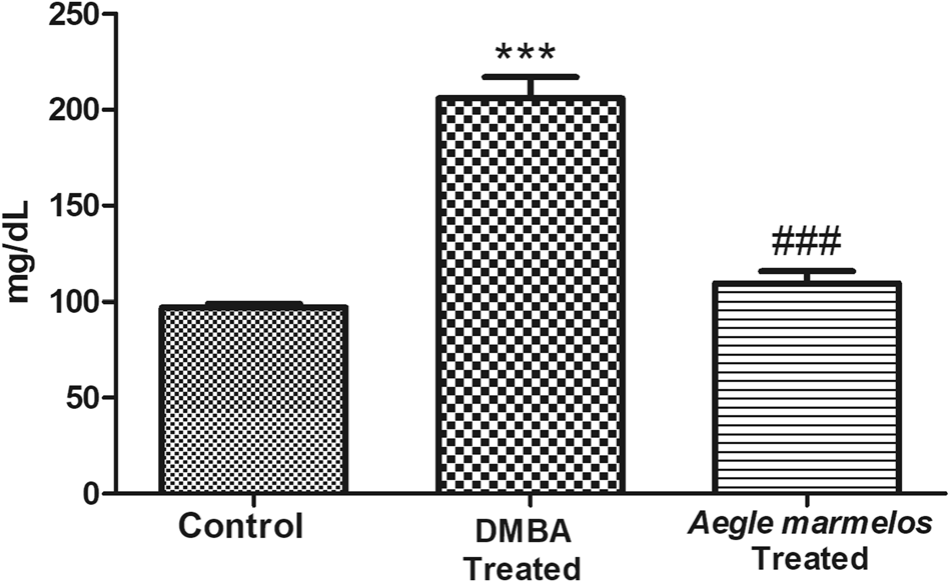
Effect of different treatments on glucose level in the studied groups (n = 4, Significant ***P < 0.0001 compared with control group, ^###^*P* < 0.0001 compared with DMBA treated group, values are expressed as mean ± SEM).

There was a significant (*P* < 0.0001) increase in blood glucose levels in the DMBA treated group as compared to the control group. However, after the administration of *Aegle marmelos* ethanolic fruit pulp extract, the blood glucose level reduced significantly (*P* < 0.0001) in comparison to the DMBA treated group (Fig. 6).

### Effect on TNF- α level

**Figure 7:**
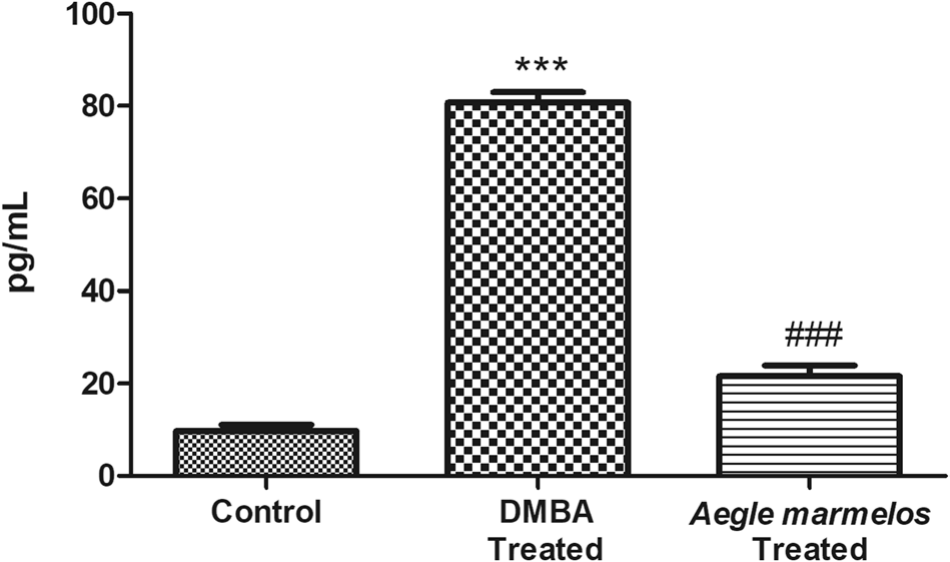
Effect of different treatments on serum TNF-α level in the studied groups (n = 4, Significant ***P < 0.0001 compared with control group, ^###^*P* < 0.0001 compared with DMBA treated group, values are expressed as mean ± SEM).

There was a significant (*P* < 0.0001) increase in the serum TNF-α levels in DMBA treated group as compared to the control group. However, after the administration of *Aegle marmelos* ethanolic fruit pulp extract, the serum TNF-α level reduced significantly (*P* < 0.0001) in comparison to the DMBA treated group (Fig. 7).

### Effect on liver biomarker parameters

The liver function biomarker parameters showed significantly (*P* < 0.0001) higher SGPT, SGOT, ALP levels and (*P* < 0.05) serum total bilirubin, and non-significant decreased levels in the serum albumin in DMBA treated group as compared to the control group. However, after the administration of *Aegle marmelos* ethanolic fruit pulp extract there was significant reduction (*P* < 0.0001) in the SGPT, SGOT and ALP levels in comparisons to the DMBA treated group (Table 1).

**Table 1:** Effect of different treatments on liver and kidney biomarker parameters in the studied groups (n = 4, Significant ***P < 0.0001, *P < 0.05 compared with control group, ^###^*P* < 0.0001 compared with DMBA treated group, values are expressed as mean ± SEM).

Parameters	Control	DMBA treated	*Aegle marmelos* treated
SGPT (U/mL)	21.01 ± 1.028	103.8 ± 2.553***	41.56 ± 2.647^###^
SGOT (U/mL)	28.90 ± 0.765	150.7 ± 6.259***	69.42 ± 5.504^###^
ALP (KA Unit)	11.31 ± 0.476	68.83 ± 1.563***	10.48 ± 1.047^###^
Total Bilirubin (mg/dL)	0.662 ± 0.035	1.170 ± 0.029*	0.907 ± 0.064
Albumin (g/dL)	3.300 ± 0.404	2.840 ± 0.563	3.510 ± 0.307
Urea (mg/dL)	21.28 ± 0.458	73.99 ± 1.526***	37.24 ± 2.740^###^
Uric acid (mg/dL)	5.56 ± 0.456	13.59 ± 0.604***	3.92 ± 0.316^###^
Creatinine (mg/dL)	0.882 ± 0.030	2.335 ± 0.089***	1.008 ± 0.097^###^

### Effect on kidney biomarker parameters

The kidney function biomarker parameters showed significant (*P* < 0.0001) higher serum creatinine, urea and uric acid levels in the DMBA treated group as compared to the control group. However, the administration of *Aegle marmelos* ethanolic fruit pulp extract significantly reduced (*P* < 0.0001) the creatinine, urea and uric acid levels in comparison to the DMBA treated group (Table 1).

### Histopathological findings

**Figure 8:**
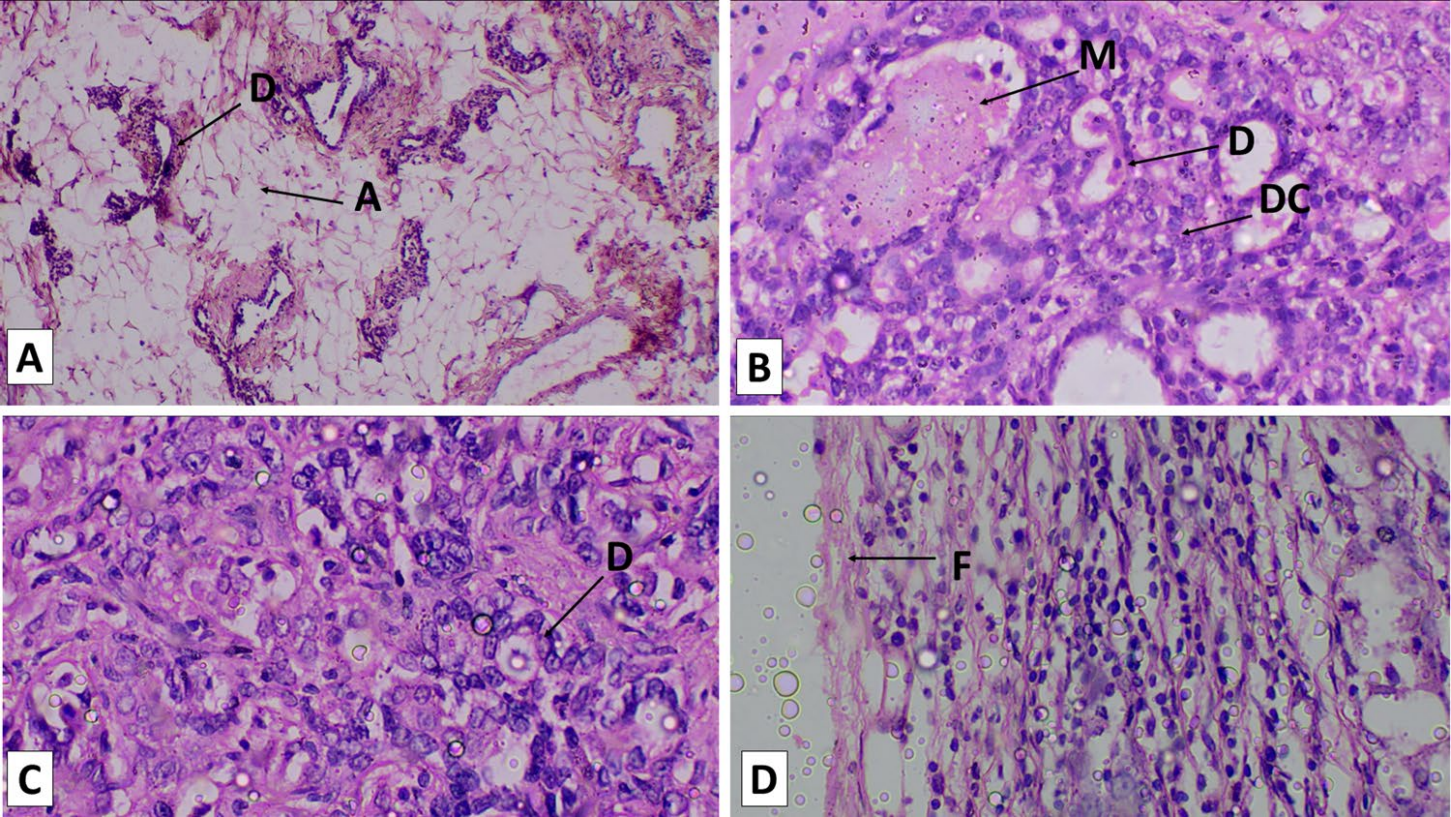
Microphotograph of rat mammary tissue stained with hematoxylin and eosin. (**A**) Section of control rat mammary tissue showing normal arrangement of adipocytes (A) & duct (D) × 500. (**B**) Mammary tissue section of DMBA treated group rat showing dedifferentiated cells (DC) with the presence of mucin (M) in the ductal (D) lumen with discontinuous basement membrane × 500. (**C**,**D**) Mammary tissue section of *Aegle marmelos* treated group rat. Although, the ducts (D) basement membrane is discontinuous but there is absence of mucin and any other foreign substances while covered with thick mass of fibrous connective tissues (F) × 500.

In the present histopathological examination, Fig. 8A, the mammary tissue section of control rat, shows normal architecture of mammary tissue. The DMBA treated group rat shows mammary tumor section Fig. 8B. The section shows presence of mucin in the ductal lumen with cytoplasm highly granulated. Some ducts revealed discontinuity in the basement membrane with papillary outgrowth of malignant cell. The degree of dedifferentiation was variable and suggestive of adenocarcinoma. The *Aegle marmelos* treated group rat shows mammary tumor section Fig. 8C,D. The tissue section represents adenocarcinoma characterised by tubular and acinar arrangement of neoplastic cells and significant pleomorphism with streaming pattern. The tumor cells were covered with thick mass of fibrous connective tissues, infiltrating with the small round nuclei of mononucleated cells. There was noticeable desmoplastic reaction characterised by presence of thick fibrous to collagen tissue reaction in tumor mass.

## Discussion

Oxidative stress with the formation of reactive oxygen species (ROS) is known to negatively affect the human health and is related to a number of human diseases including cancer. ROS alters the expression of tumor suppressor genes involved in apoptosis, increasing the expression of cytokines involved in angiogenesis, creates changes in the connections between cells and their effects on the metalloproteinase activity of proteinase involved in the metastasis^40^. The ROS also interferes with the membrane integrity of the cell by reacting with polyunsaturated fatty acid (PUFA) ultimately leading to the formation of malondialdehyde (MDA). MDA level has been widely known as an indicator of oxidative stress and antioxidant status in cancer patients^41^. DMBA the carcinogen is metabolically activated by the cytochrome p450 enzymes to the ultimate carcinogen DMBA-DE resulting in the induction of mammary cancer in rats with the formation of various ROS^14,16^. The oxidative stress resulting from the formation of ROS, negatively affects the other vital organs such as liver and kidney leading to the progression of the disease^42,43^.

In the present study, a significant reduction in the mammary tumor volume and serum TNF-α level was observed in *Aegle marmelos* treated group as compared to the DMBA treated group. The TNF-α is a proinflammatory cytokines and is also reported to be involved in tumourigenic role^44,45^. The DMBA carcinogenesis process involves TNF-α mediated increase in nuclear translocation of NF-$k$B (nuclear factor-$k$B) a transcription factor responsible for survival and proliferation of neoplastic cell^46,47^. The in vivo study, also showed inhibition of PTEN gene expression together with an increase in the expression of p-AKT in the DMBA treated mice^48^. The decreased serum TNF- α level and mammary tumor volume in the *Aegle marmelos* treated group significantly denotes the anti-inflammatory and anti-proliferative property of the plant extract. The phytochemicals marmelin and marmelosin present in the *Aegle marmelos* fruit pulp may be responsible for the observed pharmacological properties of the plant extract. These phytochemicals functions through the suppression of NF-$k$B activation and subsequent reduction in the p-AKT levels and thus decreases the cell survival, proliferation, and invasiveness^49–51^. The apoptotic pathway has also been reported to be activated by the marmelin through the activation of cascade series of caspase-8, caspase-3 and truncated Bid. The apoptotic efficiency of marmelosin has also been reported by Pynam and Dharmesh^51^. The β-caryophyllene and Caryophyllene oxide obtained from fruit extracts of *Aegle marmelos* coaxed apoptosis in Jurkat cell lines^52^. The expression of vascular endothelial growth factor (VEGF) and interleukin-8 (IL-8) that regulates capillary growth in the tumor have also been found to be reduced by marmelin treatment^17,49,50^. All of these apoptotic, anti-angiogenic and anti-inflammatory properties of the different phytochemicals may have contributed to the observed anti-proliferative property of the *Aegle marmelos* ethanolic fruit extract. The results are in line with the previous studies reported by the several researchers who supported the anti-inflammatory and anti-cancerous properties of the *Aegle marmelos* plant extract. The decreased expression of TNF-α was also reported after treatment with *Aegle marmelos* ethanolic fruit extract in rat model^53^. The anti-inflammatory property of *Aegle marmelos* fruit extract has also been validated by its protective effects against chemical induced experimental acute colitis in murine models^54–56^. Sushma and Devi^57^ have also reported the anti-genotoxic effects of *Aegle marmelos* fruit extract against cyclophosphamide induced chromosomal aberrations in Swiss albino mice. There are reports of chemopreventive potential of *Aegle marmelos* fruit extract against DMBA induced skin papillomagenesis in mice^58,59^. Moongkarndi et al.^26^ have also demonstrated the cytotoxic activity of *Aegle marmelos* ethanolic fruit extract against SKBR3 human breast adenocarcinoma cell lines. Apart from fruits, leaves of *Aegle marmelos* also contains various other phytochemicals such as lupeol, eugenol, citral, cineole, and limonene which imparts antineoplastic property to them^17,50,60^.

The extract with one of its phytochemicals, Lupeol increased the Era gene expression in MDA-MB-231 (ERα-negative breast cancer cells) and thus, inhibited the cell proliferation^61^. Most of the studies state that patients with ErbB2 overexpression cause lower overall survival rates in breast cancer patients in comparison to the non-over expressed ErbB2. Thus, drug targeting can be validated in HER-2/Neu/ErbB2-receptor tyrosine kinase especially by lupeol^62^. Furthermore, lupeol also induces apoptosis by downregulating Bcl2 (an apoptotic protein) and upregulating the Bax (a pro apoptotic protein), activating the cascade series including apaf1 gene and induces poly (ADP) ribose polymerase cleavage in the CWR22Rnu1 and PC-3 neoplastic cells^63,64^.

A significant decrease in the MDA levels in *Aegle marmelos* treated group compared to the DMBA treated group was observed. The decreased MDA levels is suggestive of inhibition of lipid peroxidation (LPO) and improved oxidative stress in the rats. The inhibition of LPO may be due to the antioxidant effects of flavonoids, tannins and phenolic compounds present in the fruit extract through increasing the activities of the antioxidant enzymes^20,21,53^. Baliga et al.^65^ also observed the preventive effect of *Aegle marmelos* fruit extract on radiation induced lipid peroxidation.

The breast cancer progression depends on various factors and important among them are steroid hormones-estrogen and progesterone that binds to the receptors present on the mammary epithelial cells leading to the growth of neoplastic cells^66,67^. In our present study, we have not observed any significant changes in the estradiol and progesterone hormone levels in the diestrous phase of the estrous cycle of the rats, but the hormone levels may be altered in the other phase of the estrous cycle.

A significant increase in the serum blood glucose level was also observed in the DMBA treated group as compared to the control group. The rise in the blood glucose level was likely due to the pancreatic damage^68^. The observed higher levels of blood glucose was expected to indicate an increased requirement of glucose for rapid proliferation of cancer cells. Duan et al.^69^ and Ryu et al.^70^ have also supported the role of hyperglycemia in rapid cancer progression. However, after *Aegle marmelos* ethanolic fruit extract administration, serum blood glucose levels was significantly reduced. The reduction in blood glucose was likely due to the presence of coumarins and flavonoids present in the *Aegle marmelos* fruit extract that may have caused regeneration of the damaged pancreatic beta cells and improved the insulin secretion from the remnant pancreatic beta cells^71,72^. The decreased blood glucose level diminishes the fuel source of the rapid proliferating neoplastic cells and thus reduces the tumor growth rate.

Since, liver and kidney are the important organs of our body; disruption of normal functions of these organs greatly hampers the metabolism of various chemotherapeutic drugs and thus increases the general body toxicity. Liver is the primary organ responsible for the metabolism of xenobiotic compounds, it is also supposed to get damaged by the chemical agents. The carcinogenic metabolites and ROS formed as result of metabolism of DMBA is thought to be responsible for the liver degeneration. In the present study, significant higher levels of serum total bilirubin, SGPT, SGOT and ALP level was observed in the DMBA treated group as compared to the control group. The higher levels of serum total bilirubin, SGPT, SGOT and ALP level in the DMBA treated group is considered to be indicative of DMBA-induced hepatic damage^73^. However, after treatment with *Aegle marmelos* ethanolic fruit extract, there was significant reduction in the serum total bilirubin, SGPT, SGOT and ALP levels. The observed reduction in the serum liver biomarker levels was indicative of the hepatoprotective effect of *Aegle marmelos* ethanolic fruit extract. The flavonoids present in the *Aegle marmelos* ethanolic fruit extract could be responsible for the hepatoprotective activity through regeneration of damaged liver part^74,75^.

Kidney is an important organ responsible for the excretion of various toxic metabolic waste products. It cannot escape the detrimental effect of the toxic metabolic products of DMBA. In the present study, there was significant elevation in urea, uric acid and creatinine levels in the DMBA treated group in comparison to the control group. The elevation in the kidney biomarker levels is considered to be indicative of DMBA induced renal toxicity and renal damage^42,43,73^. However, after administration of *Aegle marmelos* ethanolic fruit extract there was significant reduction in the urea, uric acid and creatinine levels. The observed reduction in the serum kidney biomarker levels was indicative of renal protective effect of *Aegle marmelos* ethanolic fruit extract. The phenols and flavonoid present in the *Aegle marmelos* ethanolic fruit extract could be responsible for the observed renal protective effect through the regeneration of the damaged part of kidney^76,77^.

The histopathological study also confirms the anti-proliferative nature of *Aegle marmelos* ethanolic fruit extract which is evident from the difference between the degree of severity between the *Aegle marmelos* treated group and DMBA treated group. The presence of thick fibrous to collagen tissue reaction in tumor mass which is the result of desmoplastic reaction also supports the protective and anti-proliferative nature of the *Aegle marmelos* ethanolic fruit extract.

## Conclusion

Hence, taking the entire parameters result into account, it can be concluded that *Aegle marmelos* ethanolic fruit pulp extract possesses anti-proliferative activity by suppressing the breast tumors growth rate in the rat model. The plant extract also possesses hepato-renal protective effect. Therefore, it can be targeted as novel and safe anti-cancer drug against breast cancer.
